# Construction of a Digestive System Tumor Knowledge Graph Based on Chinese Electronic Medical Records: Development and Usability Study

**DOI:** 10.2196/18287

**Published:** 2020-10-07

**Authors:** Xiaolei Xiu, Qing Qian, Sizhu Wu

**Affiliations:** 1 Institute of Medical Information/Medical Library Chinese Academy of Medical Sciences & Peking Union Medical College Beijing China

**Keywords:** Chinese electronic medical records, knowledge graph, digestive system tumor, graph evaluation

## Abstract

**Background:**

With the increasing incidences and mortality of digestive system tumor diseases in China, ways to use clinical experience data in Chinese electronic medical records (CEMRs) to determine potentially effective relationships between diagnosis and treatment have become a priority. As an important part of artificial intelligence, a knowledge graph is a powerful tool for information processing and knowledge organization that provides an ideal means to solve this problem.

**Objective:**

This study aimed to construct a semantic-driven digestive system tumor knowledge graph (DSTKG) to represent the knowledge in CEMRs with fine granularity and semantics.

**Methods:**

This paper focuses on the knowledge graph schema and semantic relationships that were the main challenges for constructing a Chinese tumor knowledge graph. The DSTKG was developed through a multistep procedure. As an initial step, a complete DSTKG construction framework based on CEMRs was proposed. Then, this research built a knowledge graph schema containing 7 classes and 16 kinds of semantic relationships and accomplished the DSTKG by knowledge extraction, named entity linking, and drawing the knowledge graph. Finally, the quality of the DSTKG was evaluated from 3 aspects: data layer, schema layer, and application layer.

**Results:**

Experts agreed that the DSTKG was good overall (mean score 4.20). Especially for the aspects of “rationality of schema structure,” “scalability,” and “readability of results,” the DSTKG performed well, with scores of 4.72, 4.67, and 4.69, respectively, which were much higher than the average. However, the small amount of data in the DSTKG negatively affected its “practicability” score. Compared with other Chinese tumor knowledge graphs, the DSTKG can represent more granular entities, properties, and semantic relationships. In addition, the DSTKG was flexible, allowing personalized customization to meet the designer's focus on specific interests in the digestive system tumor.

**Conclusions:**

We constructed a granular semantic DSTKG. It could provide guidance for the construction of a tumor knowledge graph and provide a preliminary step for the intelligent application of knowledge graphs based on CEMRs. Additional data sources and stronger research on assertion classification are needed to gain insight into the DSTKG’s potential.

## Introduction

### Background

Cancer is a leading cause of death worldwide. The International Agency for Research on Cancer estimates that there were 18.1 million new cases of cancer and 9.6 million deaths caused by cancer in 2018 [1]. Nearly 24% (4.3 million) of these cancer cases and 30% (2.9 million) of deaths occurred in China. Digestive tract cancers were responsible for 36.4% of cancer-related deaths in China, compared with <5% in both the United States and United Kingdom [2]. There is a rapid increase of digestive system cancers in China. China is currently challenged by trying to prevent and control digestive cancers.

Electronic medical records (EMRs) are digital versions of paper-based patient charts in clinician offices, clinics, and hospitals that contain detailed clinical information about the occurrence, development, and treatment of the patient’s disease [3]. They have important clinical value, such as providing data to support disease screening and prediction [4]. With the full implementation of health information technologies in China, its hospitals have accumulated large amounts of EMRs. However, the utilization rate of EMRs in China is relatively low, and research still focuses on traditional data management and statistical analysis of data from small samples [5-7]. One reason is because medical knowledge in Chinese EMRs (CEMRs) mainly exists in unstructured text, which cannot be understood by computers. In addition, medical knowledge in CEMRs is scattered; for example, the main entity type in the chief complaint field is symptom, and the main entity type in the discharge instructions field is medicine. This scattered knowledge distribution introduces obstacles to the analysis and in-depth mining of CEMRs. Furthermore, CEMRs have a unique sentence structure that is different from ordinary text, and there is no unified clinical medical terminology standard for CEMRs, which results in different clinicians using different expressions for the same medical term. For example, for the Chinese medical term “radical gastrectomy (胃癌根治术),” clinicians may write “胃癌根治” or “根治性胃癌根治术.” These characteristics of CEMRs have brought great challenges to the mining and utilization of CEMRs in the big data environment.

The knowledge graph is an emerging knowledge service technology in the era of big data and an important part of artificial intelligence [8]. It has a graph-based data structure composed of nodes (entities) and edges (semantic relations) [9]. Strengths of knowledge graphs are their abilities for information processing and knowledge organization; in addition, they can express various domain concepts and the intricate relationships between them. The knowledge graph provides an ideal technical means for connecting scattered knowledge fragments and integrating information in CEMRs. Using knowledge graph technology to organize and manage medical knowledge scattered in various parts of CEMRs can not only effectively describe and mine the relationship between medical entities and avoid information overload but also reduce the time cost for clinicians to find patient information and improve the knowledge service ability of CEMRs.

This study aimed to construct a semantic-driven digestive system tumor knowledge graph (DSTKG) based on CEMRs. Compared with previous studies, this study focused on the construction of a DSTKG schema and the representation of semantic relationships between medical concepts, with the aims of maximizing the presentation of diagnosis and treatment facts, better assisting knowledge calculation and completing knowledge graph construction, and the subsequent application of intelligent medicine. In addition, this paper introduces the characteristics of digestive system tumor diseases and tumor-related CEMRs for the purpose of improving the performance of knowledge extraction in the process of constructing a DSTKG. In the following sections, the framework of a DSTKG based on CEMRs is presented, and the construction process of a DSTKG is described in detail along with a preliminary assessment of the knowledge graph. The paper also discusses challenges and future steps.

### Related Work

The concept of the knowledge graph was formally proposed by Google in 2012 and has been popularized in academia and industry since then. Medicine is an important vertical application field of knowledge graphs. So far, there have been a large number of medical knowledge graphs, such as IBM Watson Health [10], the Partitioned Knowledge Graph built by the University of Maryland [11], and the breast cancer knowledge graph built by Huang [12]. Furthermore, researchers have also begun to study how to construct high-quality health knowledge graphs from EMRs. For instance, Rotmensch et al [13] proposed a method to automatically construct a disease-symptom knowledge graph directly from EMRs using a noisy “OR” model [13]. Kwon et al [14] used interpretable and interactive recurrent neural networks for visual analytics on EMRs. Bean et al [15] applied a knowledge graph to verify adverse drug reactions in EMRs. Medical knowledge graphs have played an important role in medical services such as information retrieval, intelligent question-and-answer, and intelligent diagnosis.

Compared with English medical knowledge graphs, the research of Chinese medical knowledge graphs is still in its infancy, especially based on CEMRs. Part of the reason is that the resources for building Chinese medical knowledge graphs are limited. For instance, there are no public Chinese medical knowledge repositories like Systematized Nomenclature of Medicine-Clinical Terms (SNOMED CT) and repositories of biomedical ontologies in Chinese like BioPortal. In addition, Chinese is different from English. Chinese has no natural space as a separator, fewer speech components, and random use of punctuation, which makes Chinese natural language processing more difficult. For CEMRs, in addition to the characteristics of Chinese that increase the difficulty of constructing knowledge graphs, their scattered knowledge distribution, unique syntax, a large number of abbreviations, and non-standard expressions of terminology further increase the difficulty of constructing Chinese medical knowledge graphs. Considering these challenges, researchers have made various attempts at creating processes for constructing Chinese medical knowledge graphs. For example, Zhang et al [16] proposed a generative model named the conditional relationship variational autoencoder to reduce the workload of data preprocessing and manual annotation of the Chinese medical corpus. Various deep learning models were used to improve the performance of named entity recognition (NER) [17-19] and relation extraction (RE) of CEMRs [20,21]. Sheng et al [22] introduced a general framework for a health knowledge graph based on cardiovascular disease EMRs. Zhou et al [23] studied the construction and application of the “knowledge-centric” knowledge graph of traditional Chinese medicine based on ancient Chinese texts. Jie et al [24] built a breast tumor knowledge graph that only contains 3 types of concepts (basic information of the patient, examination, and diagnosis) and 7 kinds of one-way semantic relationships (has_a, instance_of, attribute_of, part_of, owns, diagnosis, and detect) [24]. However, with a one-way semantic relationship, it is difficult to fully express the complex medical process of patients. For example, the semantic relationship between disease and examination is not only the examination to investigate the disease but also the examination revealing the disease. So far, some medical knowledge graphs based on CEMRs have been established, such as those for hypertension and diabetes [25,26].

However, compared with the construction of Chinese knowledge graphs in other fields, tumor knowledge graphs are still rare, especially a DSTKG. This is because the purpose of constructing a Chinese medical knowledge graph is not only to serve the grass-roots general practitioners and specialists in large hospitals but also to popularize medical knowledge for patients. Therefore, the establishment of knowledge graphs of common diseases has more extensive value in China. On the other hand, compared with common diseases, the use of knowledge graphs to assist in diagnosis of intractable diseases such as tumors has a very high dimension, so it is more difficult to construct a tumor knowledge graph. Additionally, existing works for constructing Chinese medical knowledge graphs have mainly focused on “data-centered,” namely extracting information and establishing straightforward connections [27,28]. However, they pay less attention to the schema of the knowledge graph and semantic relationships, which leads to poor conceptual standardization and scalability of the knowledge graph. With the continuous increase in the incidence and mortality of digestive system tumor diseases in China and the rapid growth of CEMR data, the construction of a DSTKG becomes urgent.

## Methods

### DSTKG Construction Framework

**Figure 1:**
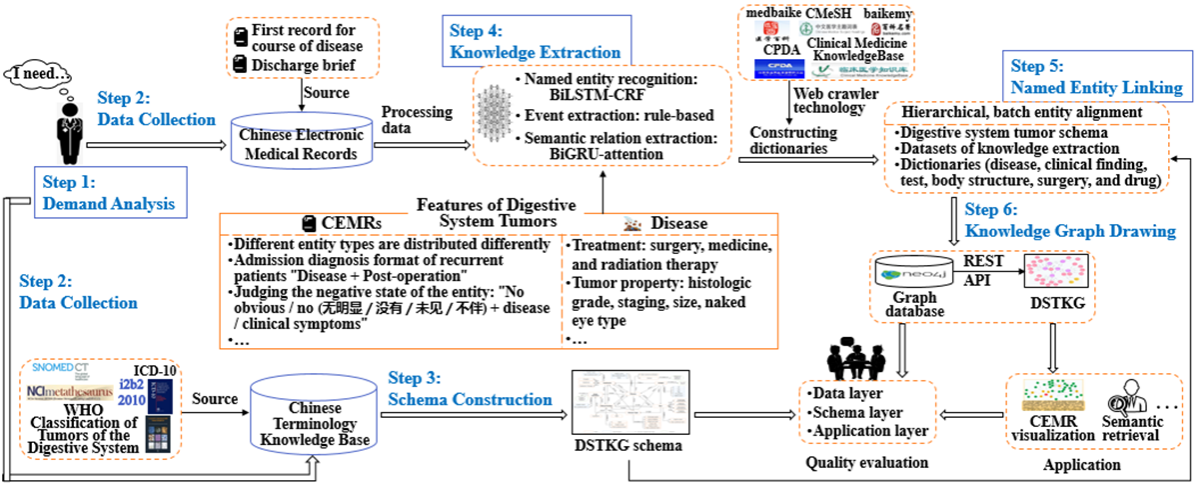
Construction framework of a digestive system tumor knowledge graph (DSTKG) based on Chinese electronic medical records (CEMRs). API: application programming interface; BiGRU: bidirectional gated recurrent unit; BiLSTM-CRF: bidirectional long short-term memory with a conditional random field; ICD: International Classification of Diseases; NCI: National Cancer Institute; REST: representational state transfer; SNOMED CT: Systematized Nomenclature of Medicine-Clinical Terms; WHO: World Health Organization.

This study takes the first record for the course of disease and the discharged brief as the data source, focusing on the construction of the DSTKG schema and enriching the types of semantic relationships between concepts. Combining the characteristics of digestive system tumor diseases and the characteristics of tumor EMRs, we designed the DSTKG construction framework based on CEMRs, as shown in Figure 1, which includes 6 steps: demand analysis, data collection, schema construction, knowledge extraction, named entity linking (NEL), and knowledge graph drawing.

Step 1 involves analyzing the construction purpose and application demands of the knowledge graph. The knowledge graph construction methods (eg, the selection of data sources and schema of the knowledge graph) would change according to the different construction purposes of knowledge graphs. First, we need to make it clear what the knowledge graph is built to do. The purpose of building a DSTKG in this study is to summarize the main contents of CEMRs as systematically and comprehensively as possible, to lay the foundation for the upper-level intelligent service.

In step 2, the data sources used to construct the knowledge graph are collected, according to the purpose of knowledge graph construction. The data sources consist of two parts: knowledge to construct the DSTKG schema and data to populate the DSTKG. CEMRs consist of 53 parts, such as medical record summary, admission record, and therapy record [29]. The patient's medical condition, diagnosis, and treatment are mainly recorded in the inpatient progress notes and discharge brief. The first record for the course of disease is the first diagnosis of the disease after admission, which is the quintessence of the inpatient progress notes. Hence, the first record for the course of disease and the discharge brief are used as the data sources for the DSTKG in this study.

Step 3 involves constructing a patient-centered DSTKG schema to organize and manage the knowledge in CEMRs. Due to the rigor of medicine, the quality of knowledge used to construct a knowledge graph schema needs to be high. We can collect domain knowledge from various existing and high-quality ontologies or terminologies, such as SNOMED CT and National Cancer Institute (NCI) Metathesaurus.

In step 4, knowledge is extracted from the data sources. This step can be divided into 3 steps: data preprocessing, NER, and semantic RE. With considerations of the diagnosis characteristics of digestive system tumor diseases and the language structure characteristics of tumor CEMRs, we employed deep learning and rule-based methods to extract knowledge from CEMRs.

After knowledge extraction, this study exploited a hierarchical and batch entity alignment strategy in step 5 to realize NEL. In the process, we constructed 6 dictionaries (eg, disease dictionary, drug dictionary) to improve the effect of NEL.

In step 6, knowledge is stored in a Neo4j graph database for preliminary feedback. After that, this study uses DSTKG for semantic retrieval and CEMR visualization. Simultaneously, this research evaluates the quality of DSTKG in order to provide ideas for further improving the knowledge graph.

### Data Sources

A knowledge graph can be divided into schema and data layers in a logical structure [30]. The schema of a knowledge graph stores refined knowledge. Accordingly, the knowledge used to construct a DSTKG schema learns from several open-access authoritative terminologies and ontologies, which are Chinese 3.4 version of SNOMED CT, the NCI Metathesaurus, World Health Organization (WHO) classification of digestive system tumors, and the second edition of the10th revision of the International Statistical Classification of Diseases and Related Health Problems (ICD-10). In addition, this study also refers to the US Informatics for Integrating Biology and the Bedside in 2010 (i2b2 2010) to determine the types of semantic relationships in DSTKG.

As for the dataset (731 CEMRs of digestive system tumors) used to build the DSTKG, it is derived from the Clinical Named Entity Recognition (CNER) challenge task of the China Conference on Knowledge Graph and Semantic Computing (CCKS) in 2018 [31]. In fact, a total of 1000 CEMRs were released by the CCKS 2018 CNER challenge task, of which 731 were CEMRs of digestive system tumors. The 731 CEMRs consist of 436 annotated CEMRs and 295 original medical records. It is worth noting that the annotated corpus only has 5 types of entities (“body structure,” “symptom,” “sign,” “surgery,” and “medicine”), which cannot meet the demands for constructing the DSTKG. Hence, under the guidance of a digestive oncology surgeon and following the principles of nonoverlapping and nonnesting, this study had 2 clinical postgraduates manually supplement 3 types of clinical entities (“disease,” “disease type,” and “test”) and 4 properties (“histological grade,” “pathological stage”, “naked eye type,” and “tumor size”) for 436 annotated CEMRs.

### Schema Construction

**Figure 2:**
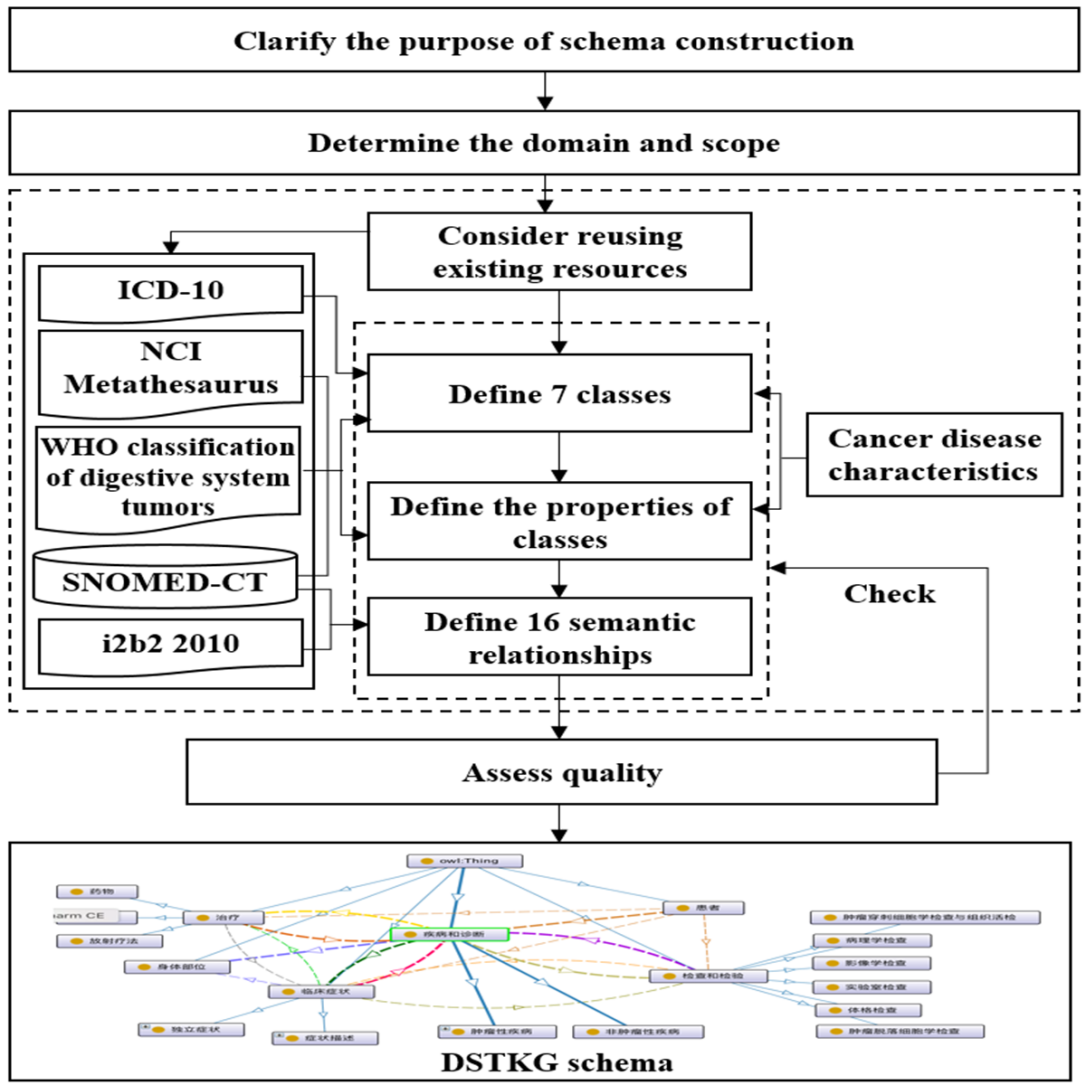
Process of constructing the digestive system tumor knowledge graph (DSTKG) schema. i2b2: Systematized Nomenclature of Medicine-Clinical Terms; ICD: International Classification of Diseases; NCI: National Cancer Institute; SNOMED CT: Systematized Nomenclature of Medicine-Clinical Terms; WHO: World Health Organization.

Lying at the core of a knowledge graph, the schema is essentially a semantic network framework that can describe knowledge normatively and objectively. This paper refers to the method of constructing an ontology by Stanford University [32]; with the help of clinical experts and ontology experts, a top-down approach was used to construct a patient-centered DSTKG schema. The Material Management System of Chinese Clinical Medical terms was utilized for schema construction. The construction process can be divided into 4 parts: clarify the purpose of schema construction, determine the domain and scope, consider reusing existing resources, and assess quality (Figure 2).

To improve the standardization and scalability of the DSTKG, we decided to build a patient-centered DSTKG schema. Based on this purpose, we determined that the field and scope of the DSTKG schema are digestive system tumors, such as stomach cancer and colorectal cancer. Then, we reused existing resources (ICD-10, NCI Metathesaurus, WHO classification of digestive system tumors, SNOMED CT, i2b2 2010) and combined the characteristics of cancer diseases to define 7 classes and their data properties, as well as 16 semantic relationships. Finally, we invited 2 ontology experts to evaluate the quality of the DSTKG schema and revise it.

The 7 classes in the DSTKG schema are “patient,” “disease,” “disease type,” “test,” “body structure,” “clinical finding,” and “treatment.” In addition, “disease” is divided into 2 subconcepts: “noncancerous disease” and “cancer.” Its knowledge source is the Chinese 3.4 version of SNOMED CT and the second edition of ICD-10. The knowledge source of “test” is the same as “disease,” which has 4 subconcepts. The classes for “clinical finding” and “body structure” have the same knowledge source, namely the Chinese 3.4 version of SNOMED CT. “Clinical finding” is divided into the 2 subconcepts of “symptom” and “sign.” “Treatment” is divided into 3 subconcepts, namely “surgery,” “medicine,” and “radiotherapy,” which are inspired by NCI Metathesaurus. Most of the concepts in “disease type” are derived from the WHO classification of digestive system tumors. In addition, each concept has its own data properties such as ID, English name, and state. More details can be found in Table 1.

**Table 1:** Data properties of the digestive system tumor knowledge graph schema.

Class	Data properties
patient	ID, sex, age, occupation, native place
disease	English name, nonpreferred term, state
disease type	English name, nonpreferred term, histological grade, pathological stage, naked eye type, tumor size
clinical finding	English name, nonpreferred term, state
test	English name, nonpreferred term
treatment	English name, nonpreferred term
body structure	English name, nonpreferred term

The semantic relationship is the representation of the semantic correlation between domain concepts that connects the concepts. We defined 16 types of semantic relationships in the DSTKG. For example, the DSTKG connects the concepts “disease” and “test” with the semantic relationship “TeCD,” which means “test is conducted to investigate the disease.” Another semantic relationship between “disease” and “test” is “TeRD,” which means “test reveals the disease.” A collection of 13 types of semantic relationships is presented in Table 2. The 3 other semantic relationships are “attribute_of,” “instance_of,” and “is_a.” Specifically, the DSTKG connects the concept with its data properties as “attribute_of” and establishes an “instance_of” relation between the concept and its entities. Further, the “is_a” relation is employed to connect the concept and its hyponym. For instance, the DSTKG connects the concepts “Primary malignant neoplasm of the stomach (胃原发性恶性肿瘤)” and “Primary malignant neoplasm of the body of the stomach (胃体原发性恶性肿瘤)” with an edge labeled “is_a” and establishes an “attribute_of” relation between the concept “Primary malignant neoplasm of the stomach (胃原发性恶性肿瘤)” and its data properties (pathological stage) “pT1N0M0.”

**Figure 3:**
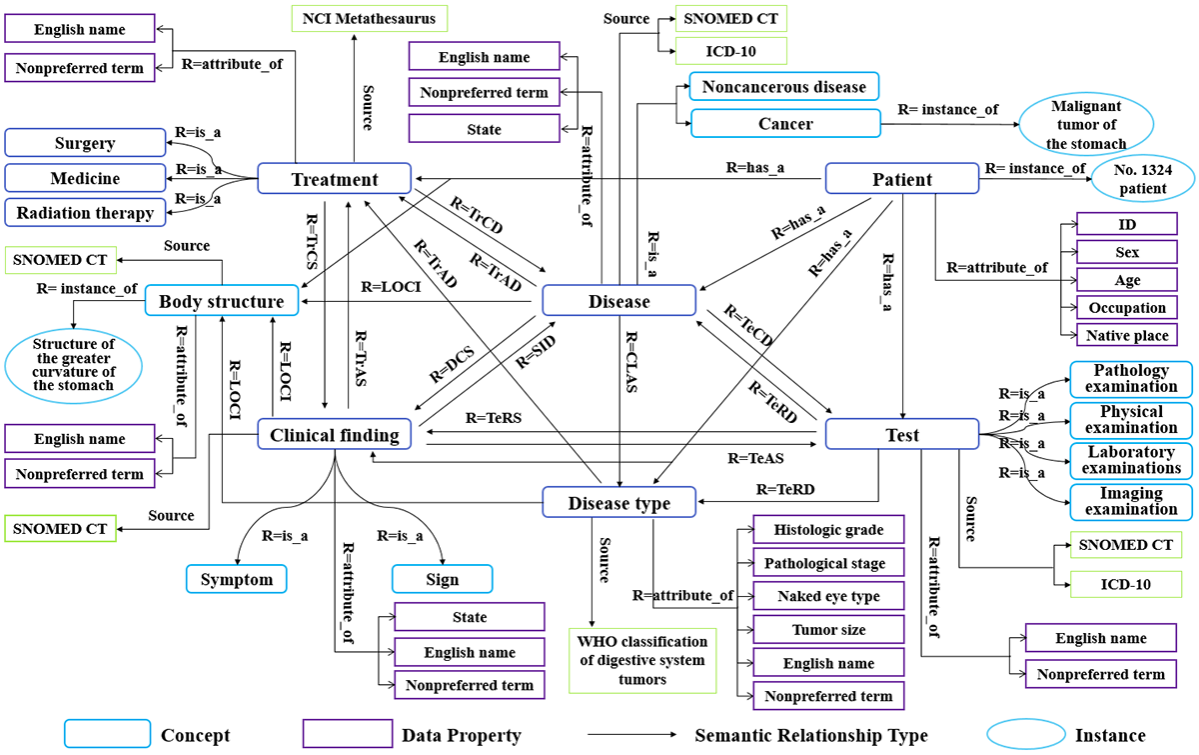
Schema of the digestive system tumor knowledge graph. CLAS: cancer disease type; DCS: disease causes symptoms and signs; ICD: International Classification of Diseases; LOCI: symptoms and signs are located in the body structure; NCI: National Cancer Institute; REST: representational state transfer; SID: symptoms and signs indicate the disease; SNOMED CT: Systematized Nomenclature of Medicine-Clinical Terms; TeCD: test is conducted to investigate the disease; TeRD: test reveals the disease; TeRS: test reveals the symptoms and signs; TrAD: treatment is administered for the disease; TrAS: treatment is administered for the symptoms and signs; TrCS: treatment causes the symptoms and signs; WHO: World Health Organization.

The schema of the DSTKG that we constructed is shown in Figure 3. This DSTKG schema basically covers the domain concept system and provides a relatively complete framework for constructing the DSTKG.

**Table 2:** Semantic relationships of the digestive system tumor knowledge graph schema.

Coarse-grained category	Semantic relationship	Definition
Test-disease relation	TeCD	Test is conducted to investigate the disease.
Test-disease relation	TeRD	Test reveals the disease.
Test-clinical finding relation	TeRS	Test reveals the symptoms and signs.
Test-clinical finding relation	TeAS	Test is administered for the symptoms and signs.
Treatment-disease relation	TrAD	Treatment is administered for the disease.
Treatment-disease relation	TrCD	Treatment causes the disease.
Treatment-clinical finding relation	TrAS	Treatment is administered for the symptoms and signs.
Treatment-clinical finding relation	TrCS	Treatment causes the symptoms and signs.
Disease-clinical finding relation	DCS	Disease causes symptoms and signs.
Disease-clinical finding relation	SID	Symptoms and signs indicate the disease.
Disease-disease type relation	CLAS	Cancer disease type
Clinical finding–body structure relation	LOCI	Symptoms and signs are located in the body structure.
Patient-disease, clinical finding, test, and treatment relation	has_a	The patient has a certain disease, clinical finding, test, or treatment.

### Knowledge Extraction

Knowledge extraction is the process of identifying valid, potentially useful, and ultimately understandable patterns from large data collections. It includes NER and RE [33].

#### Named Entity Recognition of CEMRs

**Figure 4:**
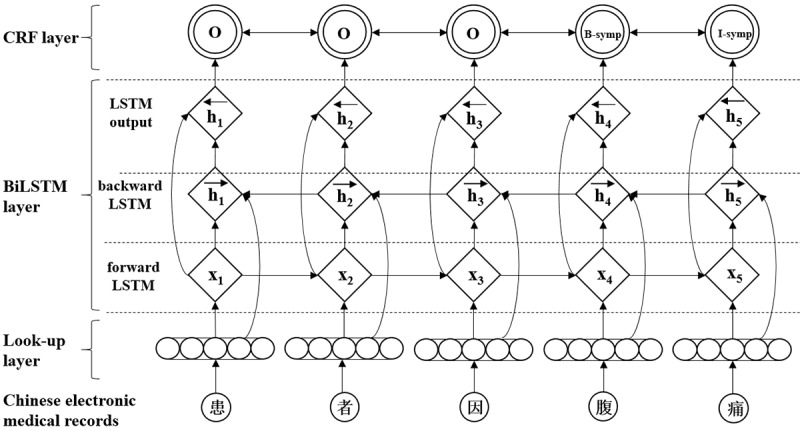
Architecture of the bidirectional long short-term memory (BiLTSM) conditional random fields (CRF). symp: symptom.

Chinese NER is usually modeled as a character-level sequence-labeling problem, because a Chinese sentence is a string of characters without an explicit delimiter [34]. For most sequence-labeling tasks, it is beneﬁcial to access both past (left) and future (right) contexts [35]. Bidirectional long short-term memory (BiLSTM) networks with a conditional random field (CRF) can do this. A BiLSTM-CRF model takes advantage of BiLSTM that can simultaneously utilize the past and future input features through the forward and backward LSTM layer. It can use the sentence-level tag information via the CRF layer as well. In terms of identifying various types of clinical entities (such as diagnoses, tests, symptoms, body part, and treatment) from CEMRs, BiLSTM-CRF achieved better performance than the baseline CRF model. Generally speaking, the F1 score of the BiLSTM-CRF model is more than 1% higher than that of the CRF model [34,36]. Therefore, this research employed a char-based BiLSTM-CRF model for NER from CEMRs. The architecture of the BiLSTM-CRF is shown in Figure 4.

The BiLSTM-CRF model consists of the look-up, BiLSTM, and CRF layers. In the look-up layer, each character in the sentence is mapped from the one-hot vector to the low-dimensional dense character vector (character embedding) as the initial input feature vector of the model. Then, the BiLSTM layer is used to extract sentence features automatically. Specifically, the forward LSTM and backward LSTM layers take the sequence of character representations X=(*x*_1_, *x*_2_,…, *x*_n_) as input and generate the representation of the left (Equation 1) and right (Equation 2) context for each character.


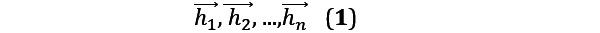



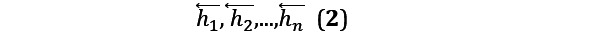


After that, the LSTM’s output layer represents the overall context sequence as 
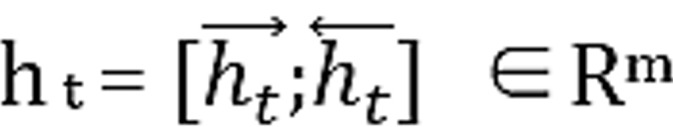
, where h_t_ is the concatenation of 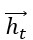 and 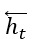


Finally, the sequence of overall context representations was taken as input for the CRF layer to predict the output label sequence.

To train the BiLSTM-CRF model, we selected 4 types of features to optimize the recognition effect: bag of characters, part-of-speech (POS), position of the character in the sentence, and dictionary features. The Natural Language Processing and Information Retrieval Chinese lexical analysis system (NLPIR-ICTCLAS) was utilized for word segmentation, while POS tags were generated simultaneously [37]. Because we used character-level information, the POS tag of the Chinese character is just the POS tag of the corresponding word that contains that character. In addition, character embeddings were learned through Gensim's word2vec on the 1000 original clinical medical records, while the segmentation information was generated by the Jieba segmentation system.

In this study, the corpus was divided into training, test, and verification sets at a ratio of 8:1:1. For the deep learning model, we set the character embedding dimension to 100, batch size of the model to 50, and learning rate to 0.0004. To alleviate the possible overfitting problem of the model, the dropout was fixed at 0.5. In the CRF model, the content window size was set to 5 to extract character features. As a result, our BiLSTM-CRF model achieved excellent overall performance with an F1 score of 0.9678, precision score of 0.9720, and recall score of 0.9636. Then, we applied the trained BiLSTM-CRF model to tag the unlabeled corpus and manually proofread the results.

For the “status” property of “clinical finding” or “disease,” we explored a rule-based approach to tag it. This is because tumor CEMRs have some fixed grammatical usage and syntactic structure. Specifically, a “clinical finding” or “disease” that indicates a negative state usually comes after “无明显／没有／未见／不伴 (not obvious/not seen/no).” For example, “患者2月余前出现上腹闷痛不适, 为饥饿时明显, 无阵发性加剧, 不伴恶心, 呕吐, 返酸, 嗳气等 (The patient started to have epigastric pain and discomfort more than 2 months ago, which was more severe when he was hungry. He has no paroxysmal aggravation, no nausea, no vomiting, no acid regurgitation, no belching, and so on.)” When the entities of “clinical finding” and “disease” with a negative property are determined, the “state” property of the remaining entities of “clinical finding” and “disease” is positive.

#### Relationship Extraction of CEMRs

RE can be simply understood as a classification problem: Given 2 entities and the sentence that appear together, distinguish the relationship between the 2 entities. Liu et al [38] found that the attention mechanism used on top of the gated regression unit (GRU) model outperforms the existing state-of-the-art neural network models on the THYME corpus in intrasentence RE from clinical narratives. Furthermore, the GRU model was also very effective on the Chinese corpus ACE2005 dataset for the entity extraction task. It only embeds Chinese character vectors, Chinese word vectors, and the regional list and can obtain high-level global features (F1=85.3) without any additional features [39]. The basic idea of the GRU model is similar to that of the LSTM model and can be regarded as a variant of LSTM. In some cases, the GRU model can produce the same excellent performance. Additionally, the GRU model can save the information in the long-term sequence and will not clear it or remove it over time because it is not related to prediction. Therefore, it can effectively solve the problem of gradient disappearance of the standard recurrent neural network [40]. Character embedding naturally adapts to Chinese characteristics. Zhou et al [41] proposed that attention-based BiLSTM memory networks outperform most of the existing methods, with only word vectors, on the SemEval-2010 relation classification task. Sentence-level attention dynamically reduces the weights of the wrong labelling problem [42]. Therefore, this paper utilizes a bidirectional GRU (BiGRU) neural network and dual-attention mechanism at the word and sentence levels to extract the relationship.

**Figure 5:**
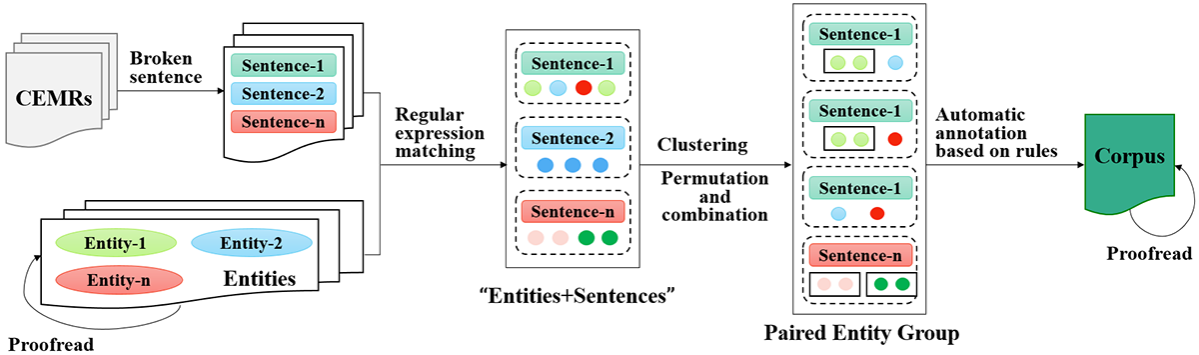
Construction process of the relational extraction corpus. CEMR: Chinese electronic medical record.

First, we performed character embedding on each Chinese character in the sentence and then fed the results to the attention GRU model at the word level. Finally, the characteristics of each sentence output are re-entered into the BiGRU, and sentence-level attention is added to solve the problem that global information cannot be expressed [43]. However, due to a lack of an annotated RE corpus at the beginning, we needed to label the corpus first, as shown in Figure 5. The materials are 731 Chinese digestive system tumor EMRs and their entity sets containing position and type information. As can be seen from Table 2, when the type of 2 entities is determined, the type of semantic relationship between them can roughly be determined too. For instance, the semantic relationship between the “disease” and “body structure” classes can only be “LOCI,” and the semantic relationship between the “disease” and “test” classes may be “TeCD” or “TeRD.” Hence, this paper first breaks the sentences in CEMRs and then uses regular expressions to match the proofread entities and sentences. In tumor CEMRs, the same types of medical entities often appear together and usually have the same semantic relationship with other types of medical entities. Based on this characteristic of tumor CEMRs, we cluster, permute, and combine the clinical entities in a sentence to make sure there are at most 2 types of entities.

In the end, according to the aforementioned rules, the RE corpus is labeled automatically and proofread manually. In this research, we annotated 45,607 sentences, of which 80% were used as training data, and the rest were used as a testing set. Moreover, BiGRU-attention achieved good performance with an F1 score of 0.5167.

### Named Entity Linking

There is usually a synonymy problem in the process of extracting knowledge from a single data source; that is, an entity has many different entity mentions, such as names, aliases, abbreviations, and even misrepresentations. NEL is an important method to solve the problem of entity ambiguity by mapping entity mentions to standard concepts in the knowledge base [44].

**Figure 6:**
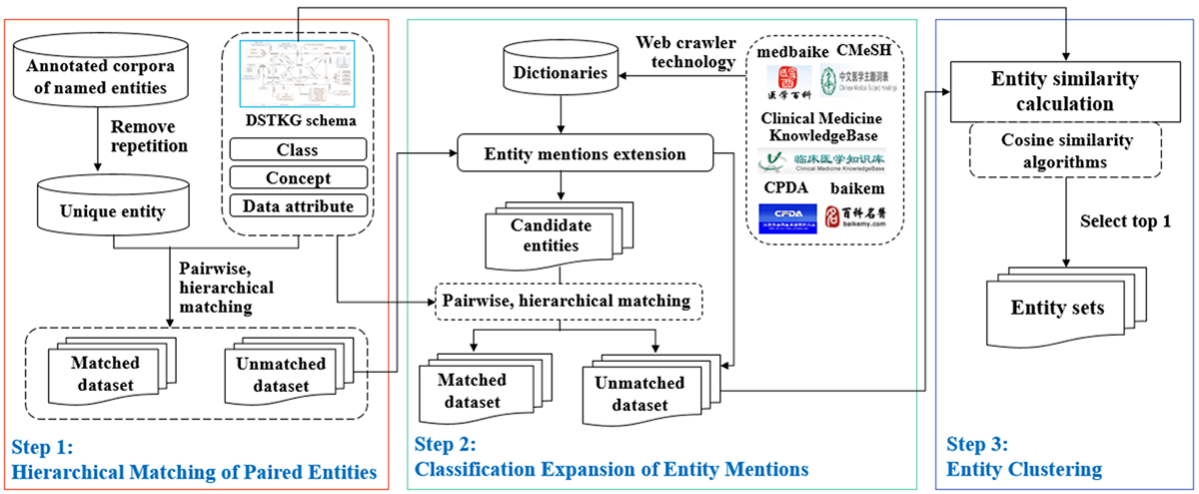
Process of hierarchical named entity linking. DSTKG: digestive system tumor knowledge base.

Before carrying out NEL, we constructed 6 dictionaries based on sources such as the State Food and Drug Administration and clinical medical knowledge base. Then, we proposed a hierarchical and batch NEL method based on the DSTKG schema, as illustrated in Figure 6. The process can be divided into 3 steps. The first step is to remove the duplicate entities of each EMR text and match them hierarchically between entities and schema. Specifically, the semantic type of the entity first matches the class; then, the entity exactly matches concepts of this class. If there is no match, an exact match will be made later on the data properties of these concepts. The second step is to use the constructed dictionaries to expand those unmatched entity mentions and repeat the step of hierarchical matching. Last, we used the rank-based approach of cosine similarity to sort those unmatched entities and select the top-ranking entities as the NEL result after disambiguation.

Eventually, the research obtained 9868 entities: 1002 “disease,” 452 “disease type,” 1874 “body structure,” 1606 “treatment,” 2786 “clinical finding,” 924 “test,” and 1224 “properties of disease type.” We also obtained 11,005 semantic relationships (Table 3).

**Table 3:** Number of each semantic relationship (N=11,005).

Semantic relationship	Definition	Number
TeCD	Test is conducted to investigate the disease.	96
TeRD	Test reveals the disease.	990
TeRS	Test reveals the symptoms and signs.	2035
TeAS	Test is administered for the symptoms and signs.	486
TrAD	Treatment is administered for the disease.	980
TrCD	Treatment causes the disease.	2
TrAS	Treatment is administered for the symptoms and signs.	613
TrCS	Treatment causes the symptoms and signs.	326
DCS	Disease causes the symptoms and signs.	29
SID	Symptoms and signs indicate the disease.	261
LOCI	Symptoms and signs are located in the body structure.	3330
attribute_of	Properties of the entity	1857

### Knowledge Graph Drawing

In this study, we chose the Neo4j graph database to draw the DSTKG. A graph database is different from a traditional relational database in that it can store the ontologically structured knowledge and visualize the relationship between entities [45]. As one of the most popular graph databases, Neo4j is an open-source database implemented in Java, which organizes data as nodes, relationships, and properties in the property graph model. Additionally, Neo4j supports an ACID-compliant transactional backend and applies Cypher for retrieving data. The syntax of Cypher is relatively simple, and its performance is not affected by the amount of data. Therefore, we used Neo4j to manage the data and draw the knowledge graph.

Considering the DSTKG’s readability, the knowledge graph first displays the top 3-tier structure of the DSTKG by default. The users can take advantage of Neo4j's node expansion to browse the DSTKG. Furthermore, the nodes at different levels are designed to have different sizes and colors for easy distinction. For example, the color of the “patient” class is red, and its nodes are larger than those of other classes, while the nodes of the “entity” are green and the smallest. Similarly, different types of semantic relationships in the DSTKG have different colors (ie, “instance_of” is orange, “TeRS” is pink, and “TrAD” is green). Finally, the DSTKG constructed in this study contains 11,372 entities and 19,276 semantic relationships [46].

## Results

After completion of the DSTKG, we used it to browse clinical knowledge and visualize CEMRs. A preliminary evaluation of the DSTKG was also carried out.

### Semantic Retrieval and CEMR Visualization

**Figure 7:**
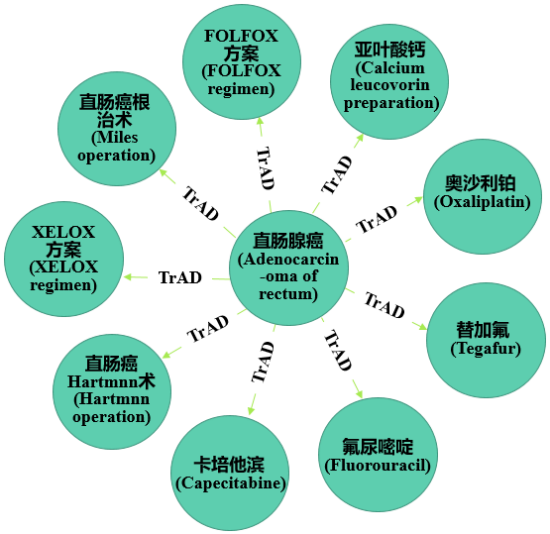
Treatment of rectal adenocarcinoma based on a search of Chinese electronic medical records using the search “MATCH (n:entities)-[r:TrAD]->(c) WHERE n.name='直肠腺癌' RETURN n,c LIMIT 2500” in the digestive system tumor knowledge graph. TrAD: treatment is administered for the disease.

The knowledge graph implements a transition from a “text-centered” retrieval model to a “things-centered” retrieval model. The DSTKG integrated 731 patients’ CEMRs so we can retrieve global or individual patient data. For instance, if you want to study the treatment of rectal adenocarcinoma (直肠腺癌), you could use the following MATCH statement: MATCH (n:entities)-[r:TrAD]->(c) WHERE n.name='直肠腺癌' RETURN n,c LIMIT 2500. The retrieval results are shown in Figure 7.

As is shown in the retrieval results of the 731 CEMRs (Figure 7), rectal adenocarcinoma had 9 treatments. Moreover, if you want to find the most frequently used treatment, you only need to perform a simple statistical analysis of the query results: 50% (365/731) of patients with rectal adenocarcinoma underwent a Miles operation, 15% (110/731) were administered oxaliplatin, 11% (80/731) of patients received the XELOX regimen, 8% (58/731) were administered capecitabine, 6% (44/731) were administered tegafur, 4% (29/731) were administered the calcium leucovorin preparation, and only 2% (15/731) each were treated with fluorouracil, the FOLFOX regimen, or a Hartmann operation.

The Miles operation, as a radical operation for lower rectal cancer and anal carcinoma, is widely used in the clinic. However, the Hartmann operation is generally performed in patients because of poor general condition, an inability to tolerate the Miles operation, or the Dixon operation is not suitable due to acute obstruction. The statistical results of this study are consistent with the actual clinical situation. Moreover, the FOLFOX regimen is also a common chemotherapy regimen for gastrointestinal tumors. The drugs included in this regimen are oxaliplatin, calcium leucovorin preparation, and fluorouracil. The reason for the small proportion of FOLFOX regimen may be due to the different writing habits of doctors. Some doctors write the FOLFOX regimen as a specific dosing program, such as oxaliplatin 85 mg/m^2^ iv gtt (2 h) D1, calcium leucovorin preparation 400 mg/m^2^ iv gtt (2 h) D1, and fluorouracil 2400 to 3000 mg/m^2^ iv gtt (continuous 46 h) D1. This will lead to an increase in the proportion of drugs used to treat rectal adenocarcinoma (such as oxaliplatin and calcium leucovorin preparation) in the statistical results, while the proportion of the FOLFOX regimen decreases. This conjecture proved to be correct by consulting the original CEMRs. Regardless of these findings, these statistical results can provide a certain degree of data to support clinicians in developing treatment plans.

**Figure 8:**
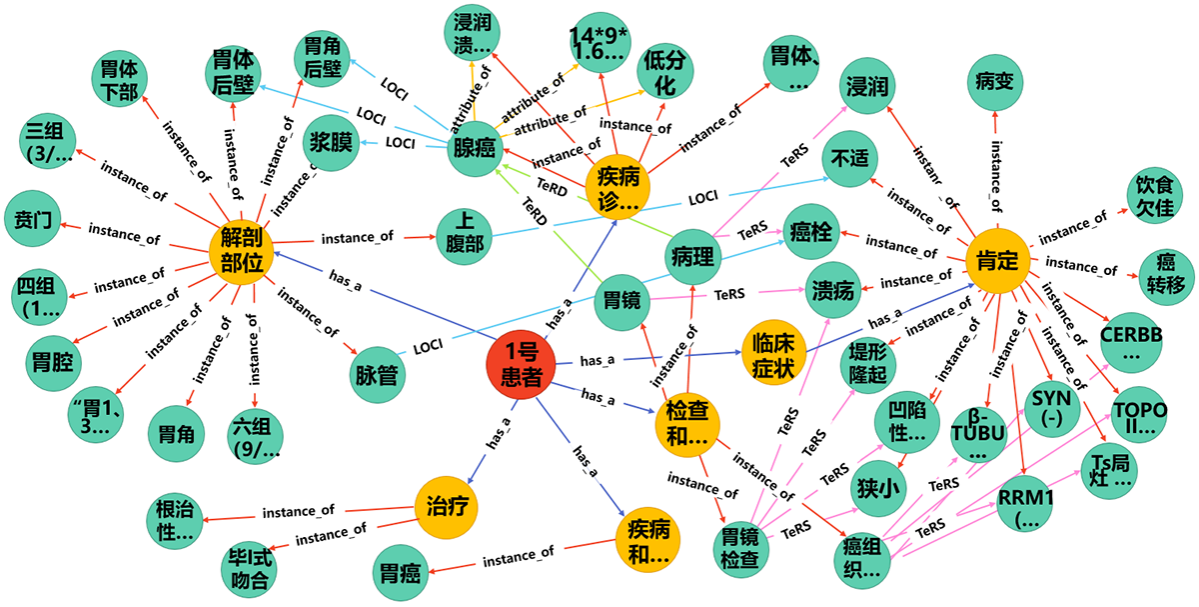
Knowledge graph of “patient no. 1.” CLAS: cancer disease type; DCS: disease causes symptoms and signs; LOCI: symptoms and signs are located in the body structure; SID: symptoms and signs indicate the disease; TeCD: test is conducted to investigate the disease; TeRD: test reveals the disease; TeRS: test reveals the symptoms and signs; TrAD: treatment is administered for the disease; TrAS: treatment is administered for the symptoms and signs; TrCS: treatment causes the symptoms and signs.

The process of disease diagnosis is generally that the doctor first understands the patient’s medical history and then issues the necessary examination documents. After the patient completes the examination, the doctor interprets the examination results and determines the treatment plan or further examination decisions. In this process, the doctor’s understanding, interpretation, and judgment of the patient’s condition are at the core of clinical work. Based on the DSTKG, we can not only view the treatment plans, clinical examinations, or common clinical findings of digestive system diseases but also query the visualization result of an individual patient's CEMRs from the DSTKG. For example, we queried the knowledge graph for “Patient no. 1” using the following statement: MATCH (n:person)-[r:has_a]->(b)-[re]->(c) WHERE n.name='1号患者' RETURN n,b,c LIMIT 2500. The visualization result is shown in Figure 8. Textbox 1 presents the original CEMR text for this patient. By comparing Figure 8 and Textbox 1, we can see that the patient's knowledge graph basically contains the main clinical entities in the CEMR text. The knowledge graph technology integrated the medical information of patient no. 1 scattered on the various working platforms of the EMR system, such as basic patient information, treatment information, and examination information and displayed them in a visual manner. With the help of the knowledge graph of patient no. 1, clinicians could grasp his main medical information at a conceptual level without having to find his information scattered on various work platforms. This not only is more intuitive but also saves time for clinicians when looking up patient medical information and effectively improves clinicians’ work efficiency. On the other hand, the integration of patient medical information can prevent clinicians from misjudgments due to omission of important patient information. Moreover, for patients, most have no medical background, and it is difficult for them to correctly find their own diagnosis and treatment information scattered in different reports. The DSTKG can provide patients with their own EMR knowledge map so they can fully understand their diagnosis and treatment process.

**The original Chinese electronic medical record text of “patient No. 1.”**

患者2个月前因上腹部不适于我院就诊, 入院后行胃镜检查示: 于胃体下部, 胃角后壁处可见较深凹陷性病变, 占胃腔1/2周, 中央可见巨大溃疡, 表面覆污苔, 周围粘膜不规则, 有明显浸润并周边呈堤形隆起; 病变部位胃腔狭小, 内镜尚能通过; 贲门部未受侵犯. 胃镜病理 (201600649), 示: (胃角, 胃体后壁) 低分化腺癌. 于2016-01-14在全麻下行根治性胃癌根治术+毕I式吻合, 术后病理示: (201600925) 胃体, 胃窦低分化腺癌, 浸润溃疡型, 体积14*9*1.6CM, 侵穿浆膜, 并于部分脉管内查见癌栓. 累及近端切线, 远端切线及另送“远端切线”未查见癌. 呈三组 (3/3个), 四组 (11/17个), 六组 (9/11个), “胃1,3,7组” (2/5个) 淋巴结癌转移. “胃第六组” (1个) 淋巴结未查见癌., 癌组织免疫组化染色示: RRM1 (-), TS局灶 (+), TOPOII部分 (+), β-TUBULIN-III (-), SYN (-), CERBB-2 (-). 术后给予静脉营养, 抑酸, 补液, 补充白蛋白, 抗感染等对症治疗. 患者恢复良好出院, 现患者为进一步行化疗来我院, 门诊以“胃癌术后”收入我科. 患者自发病以来, 精神可, 饮食欠佳, 睡眠可, 二便正常, 体重体力无明显改变.

### DSTKG Preliminary Evaluation

To appraise the reliability and practicability of the DSTKG, we adopted the expert evaluation method to preliminarily evaluate the knowledge graph from 3 aspects: data layer, schema layer, and application layer.

First, we designed a 5-level Likert scale with 9 different quality dimensions, as shown in Table 4. Then, 5 experts were invited to evaluate the quality of the DSTKG: 2 ontology experts, 1 expert in computer science, 1 urology clinical expert, and 1 hepatobiliary surgery clinical expert. At the time of expert assessment, we had not developed an interactive visualized medical knowledge service system based on the DSTKG. Hence, when the experts assessed the quality of DSTKG, we assisted them. The quality evaluation process was divided into 3 steps: (1) introducing the construction process and basic functions of the DSTKG to the experts; (2) assisting the experts to employ the DSTKG stored in Neo4j, such as retrieving specific concepts and semantic relationships or browsing the knowledge graph of specific patients; and (3) rating the DSTKG anonymously using the 5-level Likert scale. Table 4 shows the quality evaluation results.

**Table 4:** Quality evaluation score and intraclass correlation (ICC) score of the digestive system tumor knowledge graph.

Dimension and metrics		Quality evaluation score (rating)		ICC (95% CI)	
**Data layer**					
	Authority		4.73 (excellent)		0.97 (0.73 to 1.00)
	Amount of data		2.68 (adequate)		
**Schema layer**					
	Rationality		4.72 (excellent)		0.23 (–0.19 to 1.00)
	Scalability		4.67 (excellent)		
**Application layer**					
	Data consistency		4.46 (very good)		0.76 (0.41 to 0.97)
	Ease of use		3.69 (adequate)		
	Readability of results		4.69 (excellent)		
	Accuracy		4.57 (very good)		
	Practicability		3.56 (adequate)		

The mean quality evaluation score of the 5 experts was 4.20, indicating that the experts thought the DSTKG was generally good. In addition, the scores of the 2 metrics in the schema layer were higher than 4.60, which was much higher than the average. This indicated that the DSTKG schema had good performance, had reasonable structure, and was easy to expand. At the same time, the scores of “readability of results” and “accuracy” were >4.50. This could be attributed to the good performance of knowledge extraction and the NEL model, as well as the visual design of the DSTKG. However, the metric “amount of data” was scored the lowest, at 2.89, and the scores of “ease of use” and “practicability” were both <3.70. The reason was that the experts thought that the number of CEMRs was insufficient, which had a certain impact on the usability of the knowledge graph and objectivity of evaluation. Furthermore, compared with other experts, the clinical experts had higher requirements for the accuracy of the DSTKG and deemed that there was still a long way to go before the DSTKG was ready for clinical application, so they gave a very low score. About the “ease of use,” although the Cypher query language is easy to learn, it is still difficult for users who do not understand programming.

This was a preliminary evaluation. However, it provided direction for improving the DSTKG and indicated the need to better understand and contemplate the application of the DSTKG beyond a solely technological perspective.

### Consistency

We performed an interrater reliability analysis using the intraclass correlation coefficient (ICC) and 95% CI. The ICC is commonly used to assess interrater reliability for ordinal, interval, and ratio variables and is suitable when 2 or more coders are used [47]. Reliability is assessed as “perfect” at an ICC value of 1.0, “excellent” at >0.81, “substantial” at 0.80-0.61, “moderate” at 0.60-0.41, “fair” at 0.40-0.21, and “slight” at <0.20 [48,49]. This analysis was conducted using SPSS version 23.0. The results are shown in Table 4.

The ICCs for the DSTKG metrics ranged from 0.23 to 0.97 (Table 4). The ICC for the 5 experts was lowest in the schema layer (0.23), but higher in the other two dimensions (application layer, 0.76; data layer, 0.97), the interrater reliability was “substantial” to “excellent.” In general, the intrareviewer item score agreement was acceptable.

## Discussion

### Principal Findings

This paper proposes a framework for building a DSTKG based on CEMRs and describes the construction of the DSTKG according to the framework. Finally, experts evaluated its quality from different dimensions. Although this DSTKG needs further refinement, it is an attempt to provide the basis for complete and consistent reporting of this rather vague area. The responses from the experts showed the DSTKG construction framework was scientific and feasible, and the DSTKG had high rationality and some practicality. However, the ICC for the 5 experts was lower in the schema layer (ICC=0.23). On the one hand, because the schema construction is highly specialized, it is difficult for nonprofessionals to understand, so its evaluation varied greatly. On the other hand, the schema of the DSTKG is built by semi-automation. Due to different professional fields, experts have different views on this. For instance, computer science experts believed that semi-automatic construction would affect the scalability of the schema, so they gave a lower score for the scalability of the schema. Our DSTKG also displayed more granular semantic relationships and scalability than previous tumor knowledge graphs. Furthermore, clinicians can grasp patients’ medical information more quickly and conveniently than by reading CEMRs, and patients can also better understand and manage their own diseases on the basis of the DSTKG. The DSTKG is expected to contribute to a better representation of CEMRs and form the basis for further semantic research of tumors.

#### Highly Granular Knowledge Extraction

As the content of CEMRs becomes more complex, highly granular knowledge extraction of CEMR text is becoming increasingly important. The property of the concept is known as “the key feature of the concept,” which can describe the concept in a holistic manner. The property description of medical concepts not only helps further narrow the scope of possible diseases but also helps to distinguish patients with similar symptoms and provide personalized treatment. Taking this into account, this research not only defined 7 common types of concepts in the process of clinical diagnosis and treatment but also used a deep learning model or rule-based method to extract conceptual properties, such as the “histological grade” of the disease type and the “state” of disease or clinical finding. Although it is a preliminary attempt, it can provide a reference for subsequent assertion classification and entity property recognition, to further improve the accuracy and practicability of the DSTKG.

Additionally, unlike the breast tumor knowledge graph, which simply defined the semantic relationships between patients and medical concepts, the DSTKG also built rich semantic relationships between medical entities. The DSTKG contained a total of 16 types of semantic relationships. It also went a step further to refine the semantic relationships between concepts. For instance, this study defined 2 types of semantic relationships between the concepts of treatment and disease: “TrAD” for “treatment is administered for the disease” and “TrCD” for “treatment causes the disease.” Highly granular semantic relationships can increase the relevance between concepts in the text. This can not only enhance the semantic interpretation of diagnostic results but also facilitate in-depth data analysis and knowledge reasoning.

#### Easy-to-Extend DSTKG

Many aspects of the DSTKG can be easily adjusted to a designer’s focus because of the schema we built. This has several advantages over existing Chinese tumor knowledge graphs. First, the DSTKG schema was based on the international standardized thesauruses, and 6 types of medical dictionaries were used, which made the instantiation of the schema easier. In addition, the concepts in the DSTKG are relatively independent. Therefore, the DSTKG allows designers to easily add or delete a class of concepts and semantic relationships to accommodate their emphasis on a subject matter. For example, if the designer only wants to construct a knowledge graph about the clinical symptoms and examinations of patients with digestive system tumors, he can directly extract the concepts of “patient,” “clinical finding,” and “examination” and their semantic relationships to construct a schema. Of course, the designer can also easily add other types of concepts such as patient basic information. There is no inherent limit to the number of concepts and semantic relationships that can be included in our schema.

### Limitations

A limitation of this study is the lack of extensive evaluation of the DSTKG. Moreover, the lack of additional data sources may pose a challenge to promoting the use of the DSTKG. In addition to adding more data sources, strengthening the research on assertion classification and further enriching the properties of entities are considered to be a necessary next step. Therefore, we plan to develop an interactive knowledge service platform based on the DSTKG in the future, so that the DSTKG can be more widely used and evaluated.

### Conclusions

Although CEMRs contain a wealth of medical knowledge, their utilization rate is very low. Knowledge graphs are an emerging knowledge organization technology that provides a novel approach for the deep mining and utilization of CEMRs. In view of this, we proposed a framework for the construction of a DSTKG based on CEMRs and realized the construction of the DSTKG. This research not only contributes to knowledge organization in the field of digestive system tumors but also paves the way for knowledge extraction based on the characteristics of digestive system tumor diseases and tumor CEMRs. More importantly, this research promotes the development of oncology research towards semantics. In addition, the DSTKG can also evolve toward the creation of an interactive knowledge service platform for further evaluation and investigation.

## Appendix

Multimedia Appendix 1 The original Chinese electronic medical record text of “patient No. 1”.

Multimedia Appendix 2 English translation of the original Chinese electronic medical record text of “patient No. 1”.

## Abbreviations

API: application programming interface
BiGRU: bidirectional gated recurrent unit
BiLSTM-CRF: bidirectional long short-term memory with a conditional random field
CCKS: China Conference on Knowledge Graph and Semantic Computing
CEMR: Chinese electronic medical record
CLAS: cancer disease type
CNER: Clinical Named Entity Recognition
DCS: disease causes symptoms and signs
EMR: electronic medical record
DSTKG: digestive system tumor knowledge graph
GRU: gated regression unit
i2b2: Informatics for Integrating Biology & the Bedside
ICC: intraclass correlation coefficient
ICD-10: The Tenth Revision of the International Statistical Classification of Diseases and Related Health Problems
LOCI: symptoms and signs are located in the body structure
NCI: National Cancer Institute
NEL: named entity linking
NER: named entity recognition
POS: part-of-speech
RE: relation extraction
REST: representational state transfer
SID: symptoms and signs indicate the disease
SNOMED CT: Systematized Nomenclature of Medicine-Clinical Terms
TeCD: test is conducted to investigate the disease
TeRD: test reveals the disease
TeRS: test reveals the symptoms and signs
TrAS: treatment is administered for the symptoms and signs
TrAD: treatment is administered for the disease
TrCS: treatment causes the symptoms and signs
WHO: World Health Organization
