# Supplementary material for: Construction of a Digestive System Tumor Knowledge Graph Based on Chinese Electronic Medical Records: Development and Usability Study
Source: JMIR Med Inform. 2020 Oct 7;8(10):e18287. doi: 10.2196/18287 (PMC7578820; doi:10.2196/18287)
Supplement: Multimedia Appendix 1 [file medinform_v8i10e18287_app1.docx]

**The original Chinese electronic medical record text of “patient No. 1”.**

| 患者2个月前因上腹部不适于我院就诊，入院后行胃镜检查示：于胃体下部、胃角后壁处可见较深凹陷性病变，占胃腔1/2周，中央可见巨大溃疡，表面覆污苔，周围粘膜不规则，有明显浸润并周边呈堤形隆起；病变部位胃腔狭小，内镜尚能通过；贲门部未受侵犯。胃镜病理（201600649），示：（胃角、胃体后壁）低分化腺癌。于2016-01-14在全麻下行根治性胃癌根治术+毕I式吻合，术后病理示：（201600925）胃体、胃窦低分化腺癌，浸润溃疡型，体积14*9*1.6CM，侵穿浆膜，并于部分脉管内查见癌栓。累及近端切线，远端切线及另送“远端切线”未查见癌。呈三组（3/3个）、四组（11/17个）、六组（9/11个）、“胃1、3、7组”（2/5个）淋巴结癌转移。 “胃第六组”（1个）淋巴结未查见癌。，癌组织免疫组化染色示：RRM1（-）、TS局灶（+）、TOPOII部分（+）、 β-TUBULIN-III（-）、SYN（-）、CERBB-2（-）。术后给予静脉营养、抑酸、补液、补充白蛋白、抗感染等对症治疗。患者恢复良好出院，现患者为进一步行化疗来我院，门诊以“胃癌术后”收入我科。 患者自发病以来，精神可，饮食欠佳，睡眠可，二便正常，体重体力无明显改变。 |
| --- |
