# Supplementary material for: Construction of a Digestive System Tumor Knowledge Graph Based on Chinese Electronic Medical Records: Development and Usability Study
Source: JMIR Med Inform. 2020 Oct 7;8(10):e18287. doi: 10.2196/18287 (PMC7578820; doi:10.2196/18287)
Supplement: Multimedia Appendix 2 [file medinform_v8i10e18287_app2.docx]

**English translation of the original Chinese electronic medical record text of “patient No. 1”.**

| The patient's upper abdomen was not suitable for treatment in our hospital 2 months ago. After admission, gastroscopy showed that deep sunken lesions could be seen in the lower part of the gastric body and the posterior wall of the gastric angle, which occupied the 1/2 week of the gastric cavity. Large ulcers could be seen in the center of the stomach, the surface was covered with moss, the surrounding mucosa was irregular, there was obvious infiltration and the periphery showed dike-shaped eminence. The gastric cavity in the lesion site was narrow and the endoscope could still pass; the cardia was not invaded. Gastroscopic pathology (201600649), showing poorly differentiated adenocarcinoma (gastric angle, posterior wall of gastric body). Radical operation for carcinoma of stomach and Billroth I anastomosis were performed under general anesthesia on January 14, 2016. The postoperative pathology showed that (201600925) poorly differentiated adenocarcinoma of gastric body and antrum, infiltrating and ulcerating type, with a volume of 14*9*1.6CM, penetrated the serosa, and tumor thrombus was found in some vessels. No cancer was found in proximal tangent, distal tangent and other "distal tangent". The cancer metastasized to lymph nodes in group 3 (3/3), group 4 (11/17), group 6 (9/11) and "stomach group 1, 3, 7" (2/5). No cancer was found in the lymph nodes of the "stomach sixth group" (1). Immunohistochemical staining showed that RRM1 (-), TS focal (+), TOPOII (+), β-TUBULIN-III (-), SYN (-), CERBB-2 (-). After operation, patients were given symptomatic treatment, such as intravenous nutrition, acid inhibition, fluid replacement, albumin supplement, anti-infection and so on. The patient recovered well and was discharged from the hospital. Now the patient came to our hospital for further chemotherapy, and the outpatient clinic was admitted to our department as "after gastric cancer operation". Since the patient's spontaneous illness, the spirit can be, the diet is poor, the sleep can be, the two stools are normal, and there is no significant change in body weight and physical strength. |
| --- |
